# World Day of Remembrance for Road Traffic Victims — November 16, 2004

**Published:** 2014-11-14

Road traffic crashes kill nearly 3,500 persons each day worldwide and injure or disable an estimated 20–50 million persons each year (1). They are the leading cause of death among young persons aged 15–29 years worldwide, and the leading cause of death among those aged ≤30 years in the United States. CDC has declared motor vehicle injuries a “winnable battle” and supports efforts at the United Nations (UN) and World Health Organization (WHO) to dedicate 2011–2020 as the Decade of Action for Road Safety (2). The Decade of Action was launched in May 2011 in more than 100 countries with the goal of preventing 5 million road traffic deaths globally by 2020.

In October 2005, the UN General Assembly adopted a resolution (3) calling for governments and nongovernmental organizations to mark the third Sunday in November each year as the World Day of Remembrance for Road Traffic Victims. This day is dedicated to remembering the many millions killed or injured in road crashes and their families and communities. This World Day of Remembrance also pays tribute to the dedicated emergency responders, police, and medical professionals who deal with the traumatic aftermath of road death and injury.

CDC, WHO, and the UN Road Safety Collaboration encourage governments and nongovernmental organizations worldwide to observe November 16, 2014, as the World Day of Remembrance to call attention to road traffic crashes, their consequences and costs, and prevention measures. The theme of this year’s World Day of Remembrance is “Speed kills: design out speeding.” Ancillary materials are available to provide organizations with action strategies to support victims and survivors (4). Guidance for persons or groups on how to plan and organize events is available from WHO at http://whqlibdoc.who.int/publications/2006/9241594527_eng.pdf. Additional information about the World Day of Remembrance is available at http://www.worlddayofremembrance.org. Additional information about CDC’s motor vehicle injury prevention activities is available at http://www.cdc.gov/motorvehiclesafety.
